# Evaluation of the injuries in earthquake victims with computed tomography

**DOI:** 10.1590/1806-9282.20231400

**Published:** 2024-01-22

**Authors:** Hinpetch Daungsupawong, Viroj Wiwanitkit

**Affiliations:** 1 Private Academic Consultant – Phonhong, Lao People’s Democratic Republic.; 2 Chandigarh University – Mohali, India.

Dear Editor,

We would like to discuss on the publication entitled “A needful, unique, and in-place evaluation of the injuries in earthquake victims with computed tomography (CT), in catastrophic disasters! The 2023 Turkey-Syria earthquakes: part II”^1^. The purpose of this study was to examine the CT findings of adult people impacted by the 2023 Turkey-Syria earthquake. The researchers looked at 768 adult individuals who had received CT imaging following the earthquake, categorizing injuries into six categories (head, thoracic, spinal, pelvic, limb, and abdomen) and taking age groups and imaging intervals into account. The findings revealed that the frequency of injuries varied across different categories. This report can confirm that the CT might be useful in investigating crush injuries during the earthquake crisis^2^.

One major limitation of this study is its dependence solely on CT scans. While CT imaging can provide useful insights into injuries, it cannot always capture the entire extent of all injuries or provide a complete knowledge of the patients’ situations. Other diagnostic approaches, such as physical examinations and laboratory tests, may have been used to supplement the CT findings and improve the accuracy of the results. In future research, it would be beneficial to broaden the scope of the study by including a larger sample size and more diagnostic tools. This could lead to a better understanding of the injuries incurred during catastrophic earthquakes and their anatomotopographic distribution among adults. Furthermore, undertaking long-term follow-up studies to assess the outcomes and complications of these injuries could add to our understanding in this field. Furthermore, investigating the impact of various factors on the type and distribution of injuries, such as the severity of the earthquake or the existence of pre-existing conditions, could provide significant insights into disaster preparedness and response strategy.
